# Electrotherapy in Oncology Rehabilitation: Current Evidence, Safety Considerations, and Future Perspectives

**DOI:** 10.3390/jcm15145548

**Published:** 2026-07-15

**Authors:** Cristina Octaviana Daia, Dan Nicolae Păduraru, Leon Radu, Iulia Lipianu, Ana Maria Bumbea

**Affiliations:** 1 Faculty of Medicine, Carol Davila University of Medicine and Pharmacy, 050474 Bucharest, Romania; iulia.lipianu@gmail.com; 2 Physical Medicine and Rehabilitation Division, Clinical CF2 Hospital, 011464 Bucharest, Romania; leomedica2005@yahoo.com; 3 Faculty of Nursing, University of Medicine and Pharmacy of Craiova, 200345 Craiova, Romania; anamaria.bumbea@umfcv.ro

**Keywords:** oncology rehabilitation, cancer rehabilitation, electrotherapy, transcutaneous electrical nerve stimulation, photobiomodulation, deep oscillations, electromagnetic field therapy

## Abstract

**Background/Objectives:** As cancer survival improves, rehabilitation has become an increasingly important component of comprehensive oncology care, addressing the growing burden of treatment-related impairments. Although electrotherapy modalities are widely used in Physical and Rehabilitation Medicine, their application in oncology remains controversial because of persistent concerns regarding safety and potential interactions with tumor biology. This narrative review critically evaluates the current evidence regarding the clinical applications, safety, contraindications, and therapeutic potential of electrotherapy modalities in oncology rehabilitation. **Methods:** A structured literature search was performed in PubMed, Scopus, and Web of Science for studies published between January 2000 and March 2026. Evidence from randomized controlled trials, observational studies, systematic reviews, meta-analyses, clinical practice guidelines, consensus statements, and narrative reviews was analyzed. **Results:** The strongest clinical evidence supports transcutaneous electrical nerve stimulation (TENS), photobiomodulation (PBM)/low-level laser therapy (LLLT), neuromuscular and functional electrical stimulation (NMES/FES), Deep Oscillation Therapy, and extracorporeal shock wave therapy (ESWT) for selected supportive indications in oncology rehabilitation. Therapeutic ultrasound, pulsed electromagnetic field therapy (PEMF), repetitive transcranial magnetic stimulation (rTMS), Multiwave Locked System (MLS) laser, and shortwave diathermy show promising but still limited clinical evidence, whereas oncology-specific evidence remains insufficient for interferential currents, diadynamic currents, high-intensity laser therapy (HILT), Super Inductive System (SIS), and transfer of capacitive and resistive energy (TECAR) therapy. **Conclusions:** Electrotherapy in oncology rehabilitation should be considered a prescription-based therapeutic intervention rather than a group of interchangeable physical modalities. As with pharmacological therapy, treatment efficacy and safety depend on selecting the appropriate modality, establishing the correct indication, and prescribing appropriate dosimetry and treatment parameters. Current evidence supports an individualized, evidence-informed approach that integrates the biological effects of each modality with the patient’s oncological status, rehabilitation goals, and overall clinical condition, with the ultimate aim of improving function, symptom control, and quality of life.

## 1. Introduction

Continuous advances in cancer prevention, diagnosis, and treatment have transformed many oncological conditions into chronic diseases, leading to substantial improvements in survival worldwide. In several high-income countries, long-term cancer survival rates now exceed 50–60%, reflecting the impact of multidisciplinary and increasingly personalized cancer care [1,2,3].

Consequently, rehabilitation has become an essential component of comprehensive oncology care, requiring early implementation from diagnosis and continuation throughout the disease trajectory to optimize functional outcomes and quality of life [2,4,5].

From a rehabilitation perspective, the management of patients with cancer requires careful consideration of the fundamental ethical principles of non-maleficence and beneficence. While concerns regarding patient safety may discourage the use of certain therapeutic modalities, excessive caution may inadvertently deprive patients of interventions capable of improving function, reducing disability, and enhancing quality of life. In this context, evidence-based guidance is essential to support clinical decision-making and ensure an appropriate balance between therapeutic safety and rehabilitation benefit [6,7].

Cancer and its treatments may result in a broad spectrum of impairments, including pain, fatigue, motor and sensory deficits, dysphagia, sphincter dysfunction, lymphedema, cognitive impairment, mood disorders, and limitations in activities of daily living, ultimately contributing to disability and reduced quality of life [2,8]. As the prevention, reduction, and management of disability constitute core objectives of Physical and Rehabilitation Medicine (PRM), the integration of individualized rehabilitation interventions throughout the cancer continuum is both justified and essential. Within this framework, electrotherapy modalities may represent valuable adjunctive interventions for symptom management, functional restoration, and the optimization of quality of life [9].

Despite the growing recognition of rehabilitation as an essential component of comprehensive cancer care, the use of electrotherapy in oncology remains controversial. Historical concerns regarding potential effects on tumor biology, disease progression, and patient safety have contributed to the cautious use of electrotherapy modalities in clinical practice, despite their established or potential role in symptom management and functional recovery [4,10,11,12]. Therefore, a critical appraisal of the available literature is needed to clarify the safety, indications, and clinical utility of electrotherapy interventions in oncology rehabilitation. Accordingly, the aim of this narrative review is to critically evaluate the current evidence regarding safety, clinical applications, contraindications, and therapeutic potential of electrotherapy modalities in oncology rehabilitation.

## 2. Materials and Methods

### 2.1. Review Design

This study was conducted as a structured narrative review to critically evaluate the current evidence regarding the mechanisms of action, clinical applications, safety, contraindications, and practical use of electrotherapy modalities in oncology rehabilitation.

Because the quantity, quality, and level of available evidence differed substantially among electrotherapy modalities, the literature search strategy and study selection process were adapted accordingly.

### 2.2. Literature Search Strategy

A structured literature search was performed using PubMed, Scopus, and Web of Science. Publications indexed between 1 January 2000 and 31 March 2026 were considered eligible.

The search was conducted in two consecutive stages. Initially, a broad search combining the terms “cancer” OR “oncology” with “electrotherapy” was performed to obtain an overview of the available literature. Subsequently, focused searches were conducted by combining the terms “cancer/oncology” with the name of each electrotherapy modality addressed in the present review, including therapeutic ultrasound, extracorporeal shock wave therapy (ESWT), transcutaneous electrical nerve stimulation (TENS), functional electrical stimulation (FES), Deep Oscillation Therapy, photobiomodulation (PBM), low-level laser therapy (LLLT), high-intensity laser therapy (HILT), Multiwave Locked System (MLS) laser, pulsed electromagnetic field therapy (PEMF), repetitive transcranial magnetic stimulation (rTMS), Super Inductive System (SIS), interferential currents (IFC), diadynamic currents (DDC), transfer of capacitive and resistive energy therapy (TECAR), and shortwave diathermy.

Table 1 summarizes the literature search strategy, whereas Table 2 presents the search results, study selection, and principal evidence retained for each electrotherapy modality.

### 2.3. Study Selection

The literature search, screening, and study selection were independently performed by two principal authors. Any discrepancies regarding study eligibility were resolved by consensus. Publications were evaluated according to their scientific relevance, methodological rigor, clinical relevance, full-text availability, and applicability to oncology rehabilitation.

When multiple publications were available, preference was given to international clinical practice guidelines, systematic reviews, meta-analyses, randomized controlled trials, and high-quality observational studies. For each electrotherapy modality, the highest available level of clinical evidence was preferentially retained.

Human clinical studies constituted the primary evidence base. Preclinical studies were included only when they provided essential information regarding biological mechanisms or treatment safety and were clearly distinguished from clinical evidence throughout the manuscript.

### 2.4. Additional Sources and Manual Search

To ensure comprehensive coverage of the topic, the electronic search was complemented by manual hand-searching of the reference lists (citation tracking).

This process identified additional peer-reviewed publications that were not retrieved by the initial electronic search but provided important methodological, historical, or technical information.

In addition, six internationally recognized reference textbooks in Electrotherapy and Physical and Rehabilitation Medicine were consulted exclusively to describe established physical principles, mechanisms of action, technical parameters, and clinical application principles. These textbooks were used solely as background references and were not considered sources of clinical evidence.

### 2.5. Eligibility Criteria

Preference was given to peer-reviewed full-text publications directly relevant to oncology rehabilitation. Duplicate publications, editorials, letters to the editor, conference abstracts without full-text publication, and non-peer-reviewed preprints were excluded. When oncology-specific clinical evidence was limited, all eligible peer-reviewed publications relevant to the objectives of the review were considered for inclusion.

**Figure 1:**
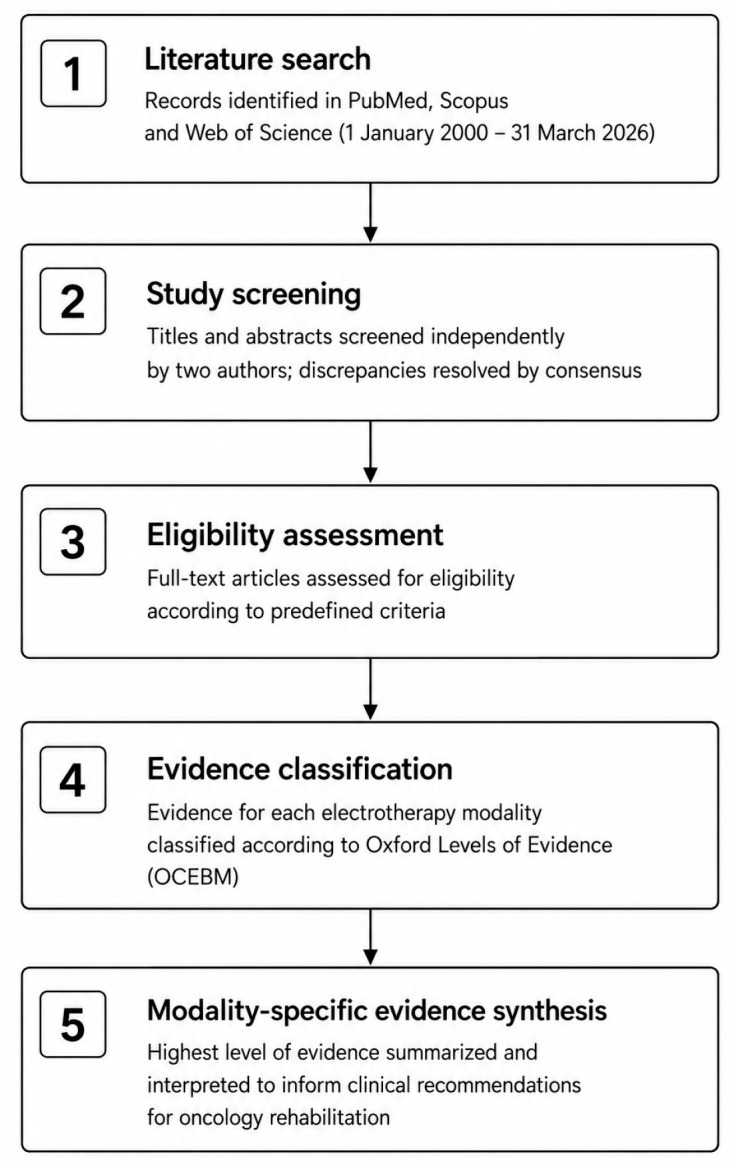
Literature search and study selection workflow.

The study selection workflow is illustrated in Figure 1.

### 2.6. Evidence Classification

The highest level of clinical evidence available for each electrotherapy modality was classified according to the Oxford Centre for Evidence-Based Medicine (OCEBM) Levels of Evidence. This framework facilitated comparison of the strength of evidence across electrotherapy modalities and supported the development of modality-specific clinical recommendations presented in this review [13].

## 3. Results

### 3.1. Contraindications and Current Challenges of Electrotherapy in Oncology Rehabilitation

Electrotherapy includes a wide range of therapeutic interventions that use electrical currents, electromagnetic fields, electromagnetic radiation, or other electrically generated physical energies to induce biological and clinical effects in target tissues [14,15,16]. Because these modalities differ in their physical characteristics, treatment parameters, and tissue effects, their appropriate prescription requires a thorough understanding of their mechanisms of action, biological effects, indications, contraindications, and dosimetry principles [12]. In PRM, electrotherapy is typically integrated into individualized rehabilitation programs. Like pharmacological interventions, these modalities produce dose-dependent effects and may be beneficial when correctly prescribed, but they may also cause adverse effects if used with inappropriate indications or treatment parameters [14,15,16].

As oncology rehabilitation evolves, the traditional contraindications and precautions associated with electrotherapy are progressively re-evaluated considering emerging clinical and experimental evidence, supporting a gradual transition from historically based restrictions toward individualized, evidence-based clinical decision-making [2,4,12]. In this context, it is important to distinguish general electrotherapy precautions from oncology-specific considerations. As in the general population, electrotherapy may be contraindicated in patients with acute illness, severe clinical decompensation, uncontrolled cardiovascular disease, implanted electronic devices, active thrombosis, or other modality-specific contraindications [17,18].

In clinical practice, contraindications should be interpreted according to both the patient’s general medical condition and the specific physical agent being prescribed. While several contraindications are common to most electrotherapy modalities, others are modality-specific or related to the oncological status of the patient [2,12,16]. 

Table 3 summarizes the general contraindications to electrotherapy together with the principal oncology-specific considerations relevant to clinical decision-making in oncology rehabilitation.

### 3.2. Oscillatory Electro-Mechanotherapy: Therapeutic Ultrasound and Extracorporeal Shock Wave Therapy

Oscillatory electromechanotherapy comprises therapeutic modalities in which electrical energy is converted into mechanical energy through electromechanical transduction mechanisms. In therapeutic ultrasound, alternating electrical current induces oscillation of a piezoelectric crystal, generating high-frequency mechanical waves that propagate through biological tissues. Similarly, extracorporeal shock wave therapy (ESWT) produces acoustic pressure waves through electrohydraulic, electromagnetic, or piezoelectric generators. Although both modalities are generally classified as mechanical physical agents, their generation fundamentally depends on electrically driven electromechanical processes.

Beyond their common physical origin, therapeutic ultrasound and ESWT share several mechanotransductive mechanisms of action. Both modalities induce biological responses through the propagation of mechanical waves within tissues, resulting in pain modulation, stimulation of tissue repair, extracellular matrix remodeling, angiogenesis, and activation of cellular signaling pathways involved in tissue regeneration. Consequently, within the conceptual framework adopted in this review and from a Physical and Rehabilitation Medicine perspective, these interventions may be considered related forms of oscillatory electromechanotherapy [19,20,21,22].

#### 3.2.1. Therapeutic Ultrasound

##### Biological Effects and Mechanisms of Action

Therapeutic ultrasound produces a variety of physical, chemical, and biological effects resulting from the propagation of high-frequency mechanical waves through biological tissues [19,23,24]. These effects include deep tissue heating, modulation of oxidation–reduction processes, phonophoresis, increased cell membrane permeability, enhanced molecular diffusion, thixotropic changes within connective tissues, acoustic microstreaming, and cavitation-related phenomena [19,24].

The biological response to therapeutic ultrasound is dose dependent. Low intensities (0.2–0.3 W/cm^2^) are primarily associated with analgesic and reflexogenic effects, whereas intermediate intensities (0.4–0.6 W/cm^2^) may promote muscle relaxation and fibrinolytic activity. Higher intensities (>0.8 W/cm^2^) generate greater mechanical stimulation and may contribute to tissue remodeling and regenerative processes [19,25].

##### Clinical Evidence and Safety Considerations for Therapeutic Ultrasound in Oncology Rehabilitation

Historically, therapeutic ultrasound has been considered contraindicated over malignant tissues because of theoretical concerns regarding tumor stimulation. However, current evidence suggests that ultrasound–tumor interactions are considerably more complex and depend on treatment parameters, tissue characteristics, and biological context. Thermal effects, mechanical stress, mechanotransduction pathways, acoustic microstreaming, and cavitation may all influence tissue responses, although their clinical significance in rehabilitation settings remains incompletely understood [26,27].

The currently available clinical evidence is limited but includes one randomized controlled trial evaluating therapeutic ultrasound as an adjunct to complex decongestive therapy in patients with breast cancer-related lymphedema. In this study, the addition of therapeutic ultrasound resulted in greater reductions in limb volume and soft-tissue thickness compared with complex decongestive therapy alone, suggesting a potential therapeutic benefit in selected patients [27].

From a clinical perspective, therapeutic ultrasound may be considered on an individual basis for selected patients presenting with soft-tissue dysfunction, pain, or muscle-related disorders, provided that significant skeletal involvement has been excluded. Low- to moderate-intensity applications (0.2–0.6 W/cm^2^) are generally preferred. Caution is required in patients with bone metastases, extensive cortical destruction, osteolytic lesions, or increased risk of pathological fracture, in whom mechanical loading may represent a greater clinical concern than the theoretical risk of tumor stimulation [26,27,28,29].

Clinical decision-making regarding therapeutic ultrasound should be based on an individualized risk-benefit assessment considering tumor location, tissue involvement, skeletal stability, rehabilitation goals, and the patient’s overall clinical condition.

Although the available evidence remains limited, the presence of a randomized controlled trial provides preliminary clinical support for therapeutic ultrasound as an adjunct to complex decongestive therapy in patients with breast cancer-related lymphedema (Oxford Level of Evidence 4). Additional well-designed randomized controlled trials are required to confirm safety, efficacy, and optimal treatment parameters in oncology rehabilitation. 

#### 3.2.2. Extracorporeal Shock Wave Therapy

##### Biological Effects and Mechanisms of Action

ESWT consists of the therapeutic application of high-pressure acoustic waves, delivered as focused or radial shock waves, which require a material medium for propagation. The biological effects of ESWT are primarily mediated through mechanical stimulation and controlled microtrauma, leading to modulation of inflammatory processes, tissue remodeling, neovascularization, and activation of cellular signaling pathways involved in tissue repair and regeneration [19,20,21,22]. 

##### Clinical Evidence and Safety Considerations for ESWT in Oncology Rehabilitation

The use of extracorporeal shock wave therapy (ESWT) in oncology has historically been approached with caution because of theoretical concerns regarding possible tumor stimulation. However, current evidence does not support considering malignancy itself an absolute contraindication. Available publications indicate that the principal contraindication is the presence of an active malignant lesion within the treatment field rather than cancer itself, provided that the treatment area is free of tumor involvement [12,30].

The available clinical evidence has expanded considerably in recent years and includes randomized controlled trials, pilot studies, and observational studies evaluating ESWT in breast cancer-related lymphedema, post-reconstruction fibrosis, pain, and functional recovery. These studies suggest that ESWT may improve pain, tissue fibrosis, limb volume, soft-tissue characteristics, and functional outcomes in selected patients, although superiority over established rehabilitation interventions, such as complex decongestive therapy, has not been consistently demonstrated [30,31,32,33,34].

The principal safety concern relates to structural tissue integrity rather than direct tumor stimulation. Consequently, ESWT should be avoided over primary tumors, active tumor masses within the treatment field, bone metastases, pathological fractures, severe cortical destruction, or other conditions associated with increased mechanical fragility. Careful assessment of skeletal stability, local tissue involvement, rehabilitation goals, and the patient’s overall clinical condition is therefore essential before treatment initiation [12,30,31,32,33,34].

Overall, the currently available evidence provides moderate clinical support for the use of ESWT in selected indications within oncology rehabilitation (Oxford Level of Evidence 2). Nevertheless, treatment should remain individualized, and further high-quality multicenter randomized controlled trials are required to define optimal treatment protocols, long-term safety, and the most appropriate clinical indications.

### 3.3. Transcutaneous Electrical Nerve Stimulation

#### 3.3.1. Biological Mechanisms of Action

Transcutaneous Electrical Nerve Stimulation (TENS) is a non-invasive electrotherapy modality primarily used for pain management. Its analgesic effects are mainly explained by the gate control theory, whereby stimulation of large-diameter sensory afferent fibers inhibits nociceptive transmission at the level of the dorsal horn of the spinal cord. Additional mechanisms include activation of descending inhibitory pathways and modulation of endogenous opioid release [35,36].

TENS can be delivered using various stimulation patterns, including conventional, symmetrical biphasic, and asymmetrical biphasic waveforms. Owing to its favorable safety profile, ease of application, low cost, and established analgesic efficacy, TENS remains one of the most widely used electrotherapy modalities in clinical practice [36].

#### 3.3.2. Clinical Evidence and Safety Considerations for TENS in Oncology Rehabilitation

The available evidence has primarily investigated the role of TENS in cancer-related pain, chemotherapy-induced peripheral neuropathy (CIPN), postoperative pain, and symptom management during supportive and palliative cancer care. Overall, the published studies suggest that TENS is a safe intervention that may provide clinically meaningful pain relief in selected patients, while no evidence currently indicates that conventional TENS promotes tumor growth or disease progression [37,38,39,40].

Although systematic reviews and randomized controlled trials have demonstrated promising results, the available literature remains limited by methodological heterogeneity, relatively small sample sizes, and variability in stimulation parameters, outcome measures, and patient populations [37,38,39,40,41].

Current evidence supports the use of TENS as a safe adjunctive modality for selected oncology patients, particularly for cancer-related pain management (Oxford Level of Evidence 1). Continued refinement of stimulation parameters, treatment protocols, and patient selection criteria may further optimize its clinical application.

### 3.4. Deep Oscillation

#### 3.4.1. Biological Effects and Mechanisms of Action

Deep Oscillation Therapy is an electrokinetic modality that combines the effects of pulsed electrostatic fields with mechanically induced tissue oscillations. Its proposed mechanisms of action include analgesic, anti-edematous, vasculotrophic, and tissue-mobilizing effects, making it a potential therapeutic option for the management of lymphedema, post-surgical edema, pain, and soft-tissue dysfunction. Owing to its minimal mechanical loading and favorable safety profile, Deep Oscillation Therapy has been investigated as a supportive intervention in rehabilitation, including cancer-related lymphedema and oncology rehabilitation [42,43,44].

#### 3.4.2. Clinical Evidence and Safety Considerations for Deep Oscillation in Oncology Rehabilitation

Available clinical studies have primarily investigated the use of Deep Oscillation Therapy for the management of cancer-related lymphedema, post-surgical edema, pain, and soft-tissue dysfunction. The published evidence indicates beneficial effects on edema reduction, symptom control, pain relief, and quality of life, particularly in patients with breast cancer-related lymphedema [42,45,46].

No evidence currently suggests that Deep Oscillation Therapy promotes tumor growth or disease progression. Consequently, it may be considered a safe supportive intervention for selected oncology patients, particularly for the management of cancer-related lymphedema (Oxford Level of Evidence 2). Further standardization of treatment dosimetry, stimulation parameters, session frequency, treatment duration, and clinical protocols is needed to facilitate evidence-based implementation in oncology rehabilitation.

### 3.5. Electrical Stimulation in Oncology Rehabilitation

#### 3.5.1. Biological Effects and Mechanisms of Action

Electrical stimulation comprises a group of electrotherapy modalities that elicit neuromuscular responses through the application of controlled electrical impulses. Depending on the degree of muscle innervation, different stimulation parameters are required. Denervated muscles respond preferentially to long-duration impulses, such as exponential or trapezoidal waveforms. Consequently, stimulation parameters should be individualized according to the underlying neurological impairment, the degree of muscle denervation, and the intended therapeutic objective [14,47].

#### 3.5.2. Clinical Evidence and Safety Considerations for Electrical Stimulation in Oncology Rehabilitation

Current evidence suggests that electrical stimulation may represent a safe and feasible intervention for selected cancer patients with sensory or motor deficits. Beneficial effects have been reported in chemotherapy-induced peripheral neuropathy, cancer-related muscle weakness, and functional impairment in hematological malignancies. Furthermore, the routine use of electrical stimulation techniques in neuro-oncology, including intraoperative cortical mapping during glioblastoma surgery, illustrates that electrical stimulation per se is not universally avoided in patients with cancer. Although these applications differ substantially from rehabilitation electrotherapy, they further challenge the concept of electrical stimulation as an absolute contraindication in oncology. Nevertheless, treatment indications and stimulation parameters should be individualized according to tumor location, neurological status, and rehabilitation goals [48,49].

The currently available evidence supports electrical stimulation as safe supportive interventions for selected oncology patients (Oxford Level of Evidence 2). Broader clinical implementation will benefit from standardized stimulation parameters, treatment dosimetry, and rehabilitation protocols.

### 3.6. Neuromuscular and Functional Electrical Stimulation (NMES/FES)

#### 3.6.1. Biological Effects and Mechanisms of Action

Neuromuscular electrical stimulation (NMES) and functional electrical stimulation (FES) are electrotherapy modalities that elicit muscle contractions through the electrical activation of intact peripheral motor nerves. Their main therapeutic objectives include the preservation of muscle mass, prevention of atrophy, improvement of muscle strength, and enhancement of functional performance [50,51,52]. Both modalities generally use short-duration rectangular pulses [14,47].

More recently, whole-body electromyostimulation (WB-EMS) has emerged as a complementary approach for improving muscle function and physical performance through simultaneous stimulation of multiple muscle groups. Although evidence regarding WB-EMS in oncology remains limited, its potential role in addressing cancer-related muscle loss and physical deconditioning warrants further investigation [18,53]. 

Owing to their potential to counteract muscle weakness and functional decline, NMES and FES have gained increasing attention as supportive interventions in oncology rehabilitation [12,47].

#### 3.6.2. Clinical Evidence and Safety Considerations for NMES/FES in Oncology Rehabilitation

Current evidence has evaluated NMES and FES primarily in patients with CIPN, cancer-related weakness, sarcopenia, hematological malignancies, and neurological impairments resulting from cancer or its treatment. Available studies indicate that electrical stimulation may enhance muscle strength, physical function, mobility, and selected sensory and motor deficits, while maintaining a favorable safety profile. 

Furthermore, the routine use of electrical stimulation techniques in neuro-oncology and neurosurgery, including cortical stimulation procedures performed during glioblastoma surgery, further illustrates that electrical stimulation per se is not universally avoided in patients with cancer. Although these applications differ substantially from rehabilitation electrotherapy, they challenge the historical concept of electrical stimulation as an absolute contraindication in oncology. Nevertheless, treatment indications and stimulation parameters should be individualized according to the underlying pathology, neurological status, and rehabilitation goals [47,48,49].

The currently available evidence supports NMES and FES as safe supportive interventions for selected oncology patients (Oxford Level of Evidence 2). Wider clinical implementation will benefit from standardized stimulation parameters, treatment dosimetry, and rehabilitation protocols.

### 3.7. Diadynamic Currents

#### 3.7.1. Biological Effects and Mechanisms of Action

Diadynamic currents (DDC) are low-frequency pulsed currents generated by the rectification of sinusoidal alternating current. Their biological effects are frequency-dependent and include neuromuscular stimulation, pain modulation, and improvement of local blood circulation. Lower frequencies predominantly produce excitatory motor responses, whereas higher frequencies are associated mainly with analgesic effects. Consequently, DDC have traditionally been used for pain management, neuromuscular stimulation, and functional rehabilitation [14,53,54].

#### 3.7.2. Clinical Evidence and Safety Considerations of DDC in Oncology Rehabilitation

Direct evidence regarding the use of DDC in oncology rehabilitation is currently lacking. No clinical studies have specifically evaluated their efficacy or safety in patients with cancer, and no evidence currently suggests tumor-promoting effects associated with this modality. Consequently, oncology-specific recommendations cannot presently be established.

The currently available evidence is insufficient to support the routine use of DDC in oncology rehabilitation (Oxford Level of Evidence: no clinical evidence). Further investigation should first determine its oncological safety before therapeutic indications and treatment protocols can be defined.

### 3.8. Interferential Current Therapy

#### 3.8.1. Biological Effects and Mechanisms of Action

Interferential current therapy (IFC) is an electrotherapy modality generated by the interaction of two medium-frequency alternating currents, producing an amplitude-modulated low-frequency current within the target tissues. Compared with conventional low-frequency stimulation, IFC is generally associated with greater patient comfort and a greater capacity to stimulate deeper tissues owing to reduced skin impedance [54,55,56,57].

Its biological effects are frequency-dependent and include neuromuscular stimulation, pain modulation, and improvement of local blood circulation. Consequently, IFC has traditionally been used for pain management, edema reduction, functional rehabilitation, and selected neurorehabilitation applications [51,52,54].

#### 3.8.2. Clinical Evidence and Safety Considerations for IFC in Oncology Rehabilitation

No oncology-specific clinical studies evaluating interferential current therapy (IFC) were identified. Consequently, the available evidence is insufficient to establish its efficacy or safety in oncology rehabilitation, and no evidence-based clinical recommendations can currently be made.

According to the Oxford Centre for Evidence-Based Medicine (OCEBM) classification, the available evidence is not classifiable because no oncology-specific clinical evidence was identified. Future clinical evaluation should first establish the oncological safety of IFC before its therapeutic role in oncology rehabilitation can be determined.

### 3.9. Photobiomodulation and Laser Therapy

#### 3.9.1. Biological Effects and Mechanisms of Action

Photobiomodulation (PBM) refers to the therapeutic use of non-ionizing light sources, including low-level laser therapy (LLLT), high-intensity laser therapy (HILT), and multiwave locked system laser therapy (MLS). These modalities deliver light energy to biological tissues, where photon absorption by intracellular chromophores, particularly within the mitochondrial respiratory chain, initiates a cascade of photochemical and photobiological responses, including increased mitochondrial activity, ATP synthesis, and modulation of cellular signaling pathways [58,59].

The biological effects of PBM include modulation of cellular metabolism, regulation of inflammatory processes, promotion of tissue repair and regeneration, and pain modulation. Depending on the wavelength, power output, and energy density delivered, laser therapies may produce predominantly photobiomodulatory effects (LLLT), combined photobiomodulatory and thermal effects (MLS), or photomechanical, photothermal, and photobiomodulatory effects (HILT) [58,60,61].

Owing to their analgesic, anti-inflammatory, and regenerative properties, laser-based therapies have been increasingly investigated in oncology rehabilitation, particularly for the prevention and management of treatment-related toxicities and functional impairments.

#### 3.9.2. Clinical Evidence and Safety Considerations for Laser Therapy in Oncology Rehabilitation

Among laser-based modalities, photobiomodulation (PBM) and low-level laser therapy (LLLT) currently have the strongest evidence base in oncology supportive care. Clinical studies, systematic reviews, and international clinical practice guidelines support their use for the prevention and management of oral mucositis associated with chemotherapy and radiotherapy, particularly in patients with head and neck cancer [62,63,64]. In a clinical series including 415 patients undergoing chemoradiotherapy for head and neck malignancies, low-dose laser therapy (1–6 J/cm^2^) was successfully applied for the prevention of oral mucositis [64].

Additional clinical evidence indicates that PBM may promote tissue repair and reduce several cancer treatment-related toxicities while maintaining a favorable safety profile when applied according to established clinical protocols [62,63,64].

Experimental studies have investigated the interaction between PBM and tumor biology. Although in vitro studies have demonstrated increased proliferation and viability in certain isolated cancer cell lines under specific irradiation conditions, these findings have not been confirmed in clinical studies. The observed biological responses appear to depend on multiple factors, including wavelength, power output, energy density, irradiation time, treatment frequency, and tumor type [65,66]. Consequently, experimental findings should not be directly extrapolated to clinical practice.

Current clinical evidence supports PBM/LLLT as a safe and effective supportive intervention for selected oncological indications, particularly for the prevention and treatment of oral mucositis, when applied according to established clinical guidelines (Oxford Level of Evidence 1). Direct irradiation of active tumors outside validated clinical indications should be avoided until further clinical evidence becomes available. 

For MLS laser, the currently available clinical evidence is limited to a single randomized controlled trial in patients with breast cancer-related lymphedema. In this study, MLS laser did not demonstrate superiority over conventional treatment or cold compression therapy, indicating that its clinical role in oncology rehabilitation remains uncertain [67]. Therefore, additional well-designed clinical trials are required before evidence-based recommendations can be established (Oxford Level of Evidence 4).

In contrast, no oncology-specific clinical evidence is currently available for high-intensity laser therapy (HILT). Consequently, its oncological safety has not been established, and no evidence-based recommendations can currently be made regarding its use in oncology rehabilitation.

### 3.10. Electromagnetic Field Therapy

#### 3.10.1. Biological Effects and Mechanisms of Action

Electromagnetic field therapies utilize time-varying magnetic fields that induce endogenous electric fields within biological tissues through electromagnetic induction. In physical and rehabilitation medicine, these modalities may be classified according to magnetic induction (B), a fundamental physical parameter governing electromagnetic energy delivery and the magnitude of the induced electric field. Accordingly, electromagnetic therapies encompass low-intensity magnetotherapy (μT–mT), pulsed electromagnetic field therapies (PEMF/rPMS; mT), transcranial magnetic stimulation (rTMS; 1–3 T), and high-intensity peripheral stimulation systems such as the Super Inductive System (SIS; up to 3 T). Their biological effects include analgesic, neuromodulatory, electrostimulatory, osteogenic, and regenerative responses [68,69].

#### 3.10.2. Clinical Evidence and Safety Considerations for Electromagnetic Field Therapies in Oncology Rehabilitation

Clinical evidence regarding electromagnetic field therapies remains limited but is expanding. A randomized controlled trial demonstrated that pulsed electromagnetic field therapy (PEMF) accelerated recovery from acute radiodermatitis in patients with breast cancer receiving radiotherapy. In addition, systematic and narrative reviews suggest potential benefits for CPIN, cancer-related pain, and supportive cancer care while maintaining a favorable safety profile [70,71,72,73,74].

In parallel, a growing body of experimental evidence indicates that PEMF may influence tumor biology through modulation of cellular proliferation, apoptosis, senescence, immune responses, and signaling pathways involved in cancer progression. Several preclinical studies have demonstrated reduced tumor cell viability, induction of apoptosis and senescence, modulation of antitumor immune activity, and enhanced sensitivity to anticancer therapies under experimental conditions [75,76]. However, these findings have not been confirmed in human clinical studies and should not be directly extrapolated to clinical practice.

The currently available clinical evidence provides preliminary support for the use of electromagnetic field therapies in selected supportive indications in oncology rehabilitation (Oxford Level of Evidence 4). At present, there is no clinical evidence supporting their use as antineoplastic therapies. Further well-designed randomized controlled trials are required to establish their safety, therapeutic indications, optimal treatment parameters, and standardized treatment protocols before broader clinical implementation.

### 3.11. Shortwave and TECAR Diathermy

#### 3.11.1. Biological Effects and Mechanisms of Action

Shortwave diathermy and transfer of capacitive and resistive energy (TECAR) are electromagnetic diathermy modalities that deliver radiofrequency energy to biological tissues. Shortwave diathermy utilizes electromagnetic waves at a standardized frequency of 27.12 MHz, whereas TECAR therapy operates at lower radiofrequencies, typically between 0.3 and 1 MHz (most commonly 448–500 kHz). Their therapeutic effects are mediated primarily through deep endogenous heating (diathermy), resulting in increased tissue metabolism, vasodilation, pain relief, muscle relaxation, and facilitation of tissue repair processes [77,78,79].

#### 3.11.2. Clinical Evidence and Safety Considerations for Shortwave and TECAR Diathermy in Oncology Rehabilitation

Shortwave diathermy and transfer of capacitive and resistive energy (TECAR) therapy have traditionally been considered contraindicated in patients with active malignancy because of concerns regarding deep tissue heating, increased local blood flow, and the theoretical possibility of influencing tumor biology. These precautions are based primarily on theoretical considerations and historical clinical practice rather than on direct clinical evidence [80].

For shortwave diathermy, oncology-specific clinical evidence remains limited. A recent proof-of-concept retrospective observational study reported the use of shortwave diathermy for the management of chronic lymphedema following breast cancer surgery, suggesting potential clinical benefits without raising specific safety concerns [81]. However, the available evidence remains insufficient to support routine clinical use, particularly over or adjacent to active tumor tissue. Consequently, shortwave diathermy should be prescribed only after an individualized risk–benefit assessment, taking into account tumor location, disease status, therapeutic objectives, and the patient’s overall clinical condition (Oxford Level of Evidence 4).

In contrast, no oncology-specific clinical studies evaluating TECAR therapy were identified. Therefore, no evidence-based recommendations can currently be made regarding its use in oncology rehabilitation. Until oncology-specific clinical evidence becomes available, TECAR therapy should be applied with caution and should generally be avoided directly over active tumor tissue (Not classifiable according to the Oxford Centre for Evidence-Based Medicine Levels of Evidence).

### 3.12. Summary of the Available Evidence and Clinical Considerations for Electrotherapy Modalities in Oncology Rehabilitation

To facilitate evidence-based clinical decision-making, Table 4 summarizes the highest available level of clinical evidence for each electrotherapy modality together with the principal supporting evidence identified in the literature.

## 4. Discussion

The available evidence indicates that electrotherapy modalities should not be considered a homogeneous therapeutic category in oncology rehabilitation. Their safety profile, biological effects, and clinical applicability differ substantially according to the type of physical energy delivered, treatment parameters, mechanism of action, anatomical site, tumor status, and patient-specific factors. Consequently, electrotherapy in oncology should be prescribed according to modality-specific evidence and individualized risk–benefit assessment rather than generalized historical contraindications [4,10,12].

Several important trends emerge from the available literature. First, TENS, PBM/LLLT, neuromuscular and functional electrical stimulation (NMES/FES), and Deep Oscillation Therapy currently have the strongest evidence base in oncology rehabilitation. Clinical studies, systematic reviews, and, for PBM/LLLT, international clinical practice guidelines support their use for selected indications, including pain management, oral mucositis, lymphedema, cancer-related weakness, sarcopenia, functional impairment, and selected manifestations of chemotherapy-induced peripheral neuropathy (CIPN) [12,40,49,63,64]. For these modalities, the principal challenge is no longer whether they may be used in selected oncology patients, but rather how treatment parameters can be standardized and integrated into individualized rehabilitation programs.

Among these modalities, PBM/LLLT represents one of the most interesting paradoxes in contemporary oncology rehabilitation. The same biological mechanisms responsible for its regenerative, anti-inflammatory, and analgesic effects—including modulation of cellular metabolism, tissue repair, and angiogenesis—have also generated theoretical concerns regarding possible tumor stimulation [65,66]. Nevertheless, clinical evidence and international guidelines consistently support PBM for selected supportive care indications, particularly the prevention and treatment of oral mucositis [63,64]. This apparent contradiction illustrates the importance of distinguishing theoretical biological concerns from clinically demonstrated therapeutic benefits.

Electromagnetic field therapies, including pulsed electromagnetic field therapy (PEMF), repetitive peripheral magnetic stimulation (rPMS), and repetitive transcranial magnetic stimulation (rTMS), represent an emerging area of oncology rehabilitation. Clinical evidence, including a recent randomized controlled trial evaluating PEMF in acute radiodermatitis, suggests potential roles in pain management, chemotherapy-induced peripheral neuropathy, neuromodulation, and supportive cancer care [71,72,73,74]. In parallel, representative preclinical studies indicate that electromagnetic stimulation may influence tumor biology through mechanisms involving apoptosis, senescence, immune modulation, and altered cellular signaling [75,76]. However, these findings remain experimental and should not be directly extrapolated to clinical practice. At present, electromagnetic field therapies should be regarded primarily as supportive rehabilitation modalities rather than antineoplastic treatments.

Although therapeutic ultrasound and extracorporeal shock wave therapy (ESWT) differ in the strength of available clinical evidence, both modalities require careful assessment of skeletal integrity and lesion location before treatment. In oncology patients, clinical decision-making should focus primarily on fracture risk, bone stability, and mechanical tissue integrity rather than generalized assumptions regarding tumor stimulation [12,26,27]. While a recent randomized controlled trial provides preliminary support for therapeutic ultrasound as an adjunct to complex decongestive therapy for breast cancer-related lymphedema, ESWT has accumulated a broader evidence base, including randomized controlled trials, pilot studies, and expert recommendations supporting its use for selected musculoskeletal conditions and breast cancer-related lymphedema [30,31,32,33,34].

For diadynamic and interferential currents, the principal limitation is the scarcity of contemporary oncology-specific evidence rather than the presence of demonstrated risks. These modalities have received little attention in modern oncology rehabilitation research, making it difficult to draw firm conclusions regarding their efficacy, safety, or optimal clinical indications. Future studies specifically designed for oncology populations are required before evidence-based recommendations can be established.

A distinct area of ongoing debate concerns shortwave diathermy and transfer of capacitive and resistive energy (TECAR) therapy. Traditionally, both modalities have been considered contraindicated in patients with active malignancy because of concerns regarding deep tissue heating, increased tissue metabolism, and local blood flow. However, these precautions are based largely on theoretical considerations rather than direct clinical evidence. A recent proof-of-concept clinical study evaluating shortwave diathermy for chronic lymphedema following breast cancer surgery suggests that this modality may warrant further clinical investigation, although the available evidence remains preliminary [81]. In contrast, no oncology-specific clinical studies evaluating TECAR therapy were identified. Therefore, conclusions regarding therapeutic heating in oncology rehabilitation should remain cautious and should not be extrapolated from oncological hyperthermia studies, which involve different treatment objectives, energy delivery systems, and multidisciplinary oncological protocols [82,83,84].

### Limitations

Several limitations should be acknowledged. First, this review is narrative in nature and does not follow a formal systematic review methodology; therefore, study selection and interpretation may be subject to selection bias despite the predefined search strategy and independent study selection.

Second, although the literature search included PubMed, Scopus, and Web of Science, relevant publications indexed outside these databases or unpublished data may not have been identified. To minimize this limitation, the electronic search was complemented by manual citation tracking and consultation of standard reference textbooks.

Third, the quantity and quality of the available evidence differed substantially among electrotherapy modalities. While TENS, PBM/LLLT, NMES/FES, Deep Oscillation Therapy, and ESWT are supported by relatively robust clinical evidence, other modalities—including therapeutic ultrasound, PEMF, MLS laser, shortwave diathermy, rTMS, HILT, DDC, IFC, SIS, and TECAR—remain supported by limited or insufficient oncology-specific evidence.

Fourth, the available studies are heterogeneous regarding cancer type, patient characteristics, intervention protocols, treatment parameters, outcome measures, and follow-up duration, limiting direct comparison across studies and precluding quantitative synthesis.

Finally, although this review primarily focuses on clinical evidence, experimental studies were included when they provided relevant information regarding biological mechanisms or treatment safety. These findings should be interpreted cautiously and should not be directly extrapolated to clinical practice without further clinical validation.

## 5. Conclusions

Current evidence demonstrates that electrotherapy modalities differ substantially in their mechanisms of action, biological effects, safety profiles, and clinical applicability in oncology rehabilitation. The strongest evidence currently supports the use of transcutaneous electrical nerve stimulation (TENS), photobiomodulation (PBM)/low-level laser therapy (LLLT), neuromuscular and functional electrical stimulation (NMES/FES), Deep Oscillation Therapy, and extracorporeal shock wave therapy (ESWT) for selected supportive indications in oncology rehabilitation. Therapeutic ultrasound, pulsed electromagnetic field therapy (PEMF), Multiwave Locked System (MLS) laser, shortwave diathermy, and repetitive transcranial magnetic stimulation (rTMS) represent promising or emerging modalities, although their clinical use is currently supported by limited evidence and requires further validation. In contrast, high-intensity laser therapy (HILT), Super Inductive System (SIS), interferential current therapy (IFC), diadynamic currents (DDC), and transfer of capacitive and resistive energy (TECAR) therapy currently lack sufficient oncology-specific clinical evidence.

The findings of this review indicate that electrotherapy should not be regarded as a homogeneous therapeutic category in oncology rehabilitation. Rather, electrotherapy should be prescribed in the same evidence-based manner as pharmacological therapy, recognizing that its biological and clinical effects are modality-specific and dose-dependent. Appropriate selection of the physical agent, treatment parameters, and dosimetry is essential to maximize therapeutic benefit while minimizing potential risks. Consequently, historical generalized contraindications should progressively be replaced by an evidence-informed approach based on individualized risk–benefit assessment and the specific characteristics of each electrotherapy modality.

Although important advances have been made in recent years, substantial knowledge gaps remain for several electrotherapy modalities. Future research should prioritize well-designed oncology-specific clinical trials to establish modality-specific recommendations, standardize treatment protocols and dosimetry, optimize treatment parameters, and further clarify the interactions between physical agent modalities, tumor biology, functional recovery, and quality of life in cancer survivors.

## Figures and Tables

**Table 1:** Literature search strategy.

Item	Description
Review design	Structured narrative review
Primary databases	PubMed, Scopus, Web of Science
Search period	1 January 2000–31 March 2026
Language	All languages
Primary evidence	Human clinical studies
Supporting evidence	Mechanistic and experimental evidence (biological mechanisms and safety only)
Search strategy	Broad search followed by modality-specific searches
Main search terms	Cancer/oncology combined with each electrotherapy modality
Additional sources	Manual citation tracking and six standard reference textbooks
Study selection	Independent evaluation by two principal authors; consensus agreement
Selection criteria	Clinical relevance, methodological quality, full-text availability, and applicability to oncology rehabilitation
Excluded publications	Duplicates, editorials, letters, conference abstracts without full text, non-peer-reviewed preprints, and publications outside the scope of the review
Reviewers	Two principal authors (independent screening and consensus selection)

**Table 2:** Literature Search Results and Principal Evidence Retained.

Search Terms	Records Identified in PubMed, Scopus and Web of Science (*n*)	Principal Evidence Retained
Cancer OR oncology AND electrotherapy	391/444/111	General reviews
Cancer OR oncology OR rehabilitation AND therapeutic ultrasound	67/160/18	Randomized controlled trial (RCT); clinical reviews
Cancer OR oncology AND extracorporeal shock wave therapy (ESWT)	22/113/23	RCT; pilot clinical studies; expert recommendations
Cancer OR oncology AND transcutaneous electrical nerve stimulation (TENS)	42/87/65	Cochrane reviews; systematic reviews; RCT
Cancer OR oncology AND functional electrical stimulation (FES)	36/69/26	Clinical studies; reviews
Cancer OR oncology AND Deep Oscillation Therapy	20/28/13	RCT; clinical studies; systematic review
Cancer OR oncology AND photobiomodulation (PBM)/low-level laser therapy (LLLT)	63/166/111	Clinical guidelines; systematic reviews; RCTs; mechanistic reviews
Cancer OR oncology AND high-intensity laser therapy (HILT)	2/3/1	No oncology-specific clinical studies
Cancer OR oncology AND Multiwave Locked System (MLS) laser	7/26/1	RCT; clinical review
Cancer OR oncology AND pulsed electromagnetic field therapy (PEMF)	13/59/25	RCT; systematic review; meta-analysis; mechanistic reviews
Cancer OR oncology AND repetitive transcranial magnetic stimulation (rTMS)	42/56/26	Clinical study; narrative reviews
Cancer OR oncology AND Super Inductive System (SIS)	1/37/122	No oncology-specific clinical studies
Cancer OR oncology AND interferential currents (IFC)	129/140/81	General review
Cancer OR oncology AND diadynamic currents (DDC)	10/26/66	No oncology-specific clinical studies
Cancer OR oncology AND shortwave diathermy	9/212/18	Observational clinical study; review
Cancer OR oncology AND transfer of capacitive and resistive energy therapy (TECAR)	15/58/50	No oncology-specific clinical studies

Note: Abbreviations: *n*, number of records; WoS, Web of Science; RCT, randomized controlled trial.

**Table 3:** General Contraindications to Electrotherapy and Oncology-Specific Considerations in Oncology Rehabilitation.

General Electrotherapy Contraindications	Oncology-Specific Considerations
Acute illness	Theoretical risk of influencing tumor biology
Severe clinical decompensation	Risk of clinical destabilization
Uncontrolled cardiovascular conditions	Tumor location and proximity to the treatment area
Implanted electronic devices	Current oncological status and disease stage
Active thrombosis or thromboembolic disease	Interaction with ongoing anticancer treatments
Pregnancy and other modality-specific contraindications	Individualized risk–benefit assessment
Severe impairment of general condition or cachexia	Need for individualized prescription and monitoring

**Table 4:** Highest level of evidence and main supporting evidence for electrotherapy modalities in oncology rehabilitation.

Electrotherapy Modality	Oxford Level of Evidence	Evidence Retained	Main Supporting Evidence
TENS	Level 1	SRs; Cochrane review; RCTs	Recommended
NMES/FES	Level 2	RCTs; reviews	Supported
Deep Oscillation	Level 2	RCTs; reviews	Supported
PBM/LLLT	Level 1	Guidelines; SRs; RCTs	Guideline-supported
Therapeutic ultrasound	Level 4	Single RCT	Limited evidence
ESWT	Level 2	RCTs; pilot studies	Supported
PEMF	Level 4	Single RCT; SR; MA	Preliminary evidence
rTMS	Level 5	Narrative reviews	Limited evidence
MLS laser	Level 4	Single RCT	Limited evidence
HILT	NC	No clinical evidence	Insufficient evidence
SIS	NC	No clinical evidence	Insufficient evidence
IFC	NC	No clinical evidence	Insufficient evidence
DDC	NC	No clinical evidence	Insufficient evidence
TECAR	NC	No clinical evidence	Insufficient evidence
Shortwave	Level 4	Single observational study	Preliminary evidence

Abbreviations: MA, meta-analysis; NC, not classifiable (no oncology-specific clinical studies identified; therefore, an Oxford Level of Evidence could not be assigned); RCT, randomized controlled trial; SR, systematic review.

## Data Availability

This study is a narrative review based exclusively on previously published literature. No new datasets were generated or analyzed during the current study. Therefore, data sharing is not applicable.

## Abbreviations

The following abbreviations are used in this manuscript:

CIPN	chemotherapy-induced peripheral neuropathy
DDC	diadynamic current
ESWT	extracorporeal shock wave therapy
FES	functional electrical stimulation
HILT	high-intensity laser therapy
IFC	interferential current
LLLT	low-level laser therapy
MA	meta-analysis
MLS	multiwave locked system laser therapy
NC	not classifiable
NMES	neuromuscular electrical stimulation
PEMF	pulsed electromagnetic field therapy
PMB	photobiomodulation
PRM	Physical and Rehabilitation Medicine
RCT	Randomized Controlled Trial
rPMS	repetitive peripheral magnetic stimulation
rTMS	repetitive transcranial magnetic stimulation
SIS	super inductive system
SR	systematic review
TECAR	transfer of capacitive and resistive energy
TENS	transcutaneous electrical nerve stimulation
WB-EMS	whole-body electromyostimulation
WOS	web of science
